# Observations on the Treatment of Stricture of the Rectum

**Published:** 1820-07

**Authors:** Charles Hicks

**Affiliations:** Surgeon.


FOR THE LONDON MEDICAL AND PHYSICAL JOURNAL.

### *Observations on the Treatment of Stricture of the Rectum.*

By Charles Hicks, Esq. Surgeon.

FROM your [i.'e. the Editor's] remarks on some observations of Mr. White, at Bath, concerning strictures, contained in your Number for April, I perceive, that the existence of a permanent simple contraction of the rectum is not universally admitted by the medical world; and that the application of the bougie in cases of this nature, is considered, even by yourselves, as an invention of recent date.* My zeal for the promotion of that science which has been the principal object of my life, forbids me to withhold whatever information it is in my power to supply on this occasion:

> Cœlicolis gratum reor, ire per omnes
> Hoc opus, et sacras populis notescere leges.

Nor do I hesitate to afford the testimony of my experience, not only as to the liability of the intestinal canal to such contractions, but to the long-established efficacy of the above-mentioned mode of practice for its removal.

Towards the close of the year 1807, during my residence at Mr. Brookes's Theatre of Anatomy, to the example of whose indefatigable industry and sagacious penetration I am so much indebted, some interesting cases of dissection came before me, which first suggested the probability of the intestinal canal being subject to simple contractions, without any apparent disease or disorganization. The importance of this discovery fixed my attention, and engaged me in a course of dissections for the investigation of its truth. The result demonstrated the fact beyond the power of refutation; and so early as in the following year produced thorough conviction in my mind as to the proper treatment of this class of diseases, however modified by variety of constitutions and symptoms incidental to general practice. I have ever since persevered with increased confidence in these original principles,—principles which develop that complex system in the generation of diseases grounded upon sympathetic origin, as opposed to mere topical affections; the study of which is the foundation of every thing scientific or widely beneficial in physiology.

The first application I made of the bougie in this species of disease, was in the summer of the year 1808, for a simple stricture of the rectum, of rather an inveterate nature, attended

* We never intended to intimate that the use of the bougie for strictures of the rectum was of recent date; and we are, besides, unable to discern which of our remarks in the critique in question will bear the interpretation given to them by Mr. Hicks.—Edit.

by affections of the head. The success which followed this experiment, (for such it certainly was at that time,) equalled my most sanguine expectations, and justified my reliance on the sole use of the bougie for all similar affections.

In the course of the last eleven years, numerous anomalous cases have been submitted to me, pronounced by practitioners of eminence to be purely idiopathic, and for a length of time ineffectually treated by them as such; but which, through the application of the bougie alone, have proved to be merely sympathetic, and have disappeared on the restoration of the intestinal functions. Some of these cases are remarkable both for magnitude and duration; and thus satisfactorily confirm the important theory of sympathetic affections dependant on the contraction of the intestinal canal, and solely removed by the restoration of its functions.

But I may probably lay some of these interesting cases before the public at my first leisure. At present, professional engagements oblige me to confine myself to the testimony of my practice towards the establishment of this very important theory, as well as of the general liability of the intestinal canal to simple contraction, without apparent disease or disorganization.

*Bath; May* 1820.

---

### COLLECTANEA MEDICA:

CONSISTING OF

### ANECDOTES, FACTS, EXTRACTS, ILLUSTRATIONS, &c.

[This part of the Journal must be vacant in the present Number, in consequence of the unusual length of the Original Communications.—EDIT.]

